# Compound Xuanju Capsule combined with western medicine for the treatment of male oligoasthenotspermia: A protocol for systematic review and meta-analysis

**DOI:** 10.1097/MD.0000000000022733

**Published:** 2020-10-16

**Authors:** Lili Chen, Hongyi Lan, Yuyan Zhang, Xiaoyan Zhang, Yu Liu

**Affiliations:** a Department of Reproductive Medicine, Yantai Yuhuangding Hospital, Affiliated Hospital of Qingdao University, Yantai, Shandong province; b Tianjin University of Traditional Chinese Medicine, Tianjin, China.

**Keywords:** compound Xuanju capsule, oligoasthenotspermia, protocol, reproductive function, systematic review

## Abstract

**Background:**

Oligoasthenotspermia is a condition in which the number and motility of sperm in the semen of fertile men are lower than the normal level. Oligoasthenotspermia not only causes damage to the reproductive system, but also causes infertility in severe cases. Compound Xuanju capsule is a kind of Chinese patent medicine. Traditional medicine believes that compound Xuanju capsule can nourish kidney Yang, benefit kidney essence, improve semen quality, and treat infertility caused by oligoasthenotspermia. Clinical practice shows that compound Xuanju capsule combined with western medicine has a good therapeutic effect on male oligoasthenotspermia, but there is no evidence of evidence-based medicine. The purpose of this study is to systematically evaluate the efficacy and safety of compound Xuanju capsule combined with western medicine in the treatment of male oligoasthenotspermia, and to improve the evidence-based basis for the clinical application of compound Xuanju capsule in the treatment of male oligoasthenotspermia.

**Methods:**

A systematic search was performed by retrieving on English database (PubMed, Embase, Web of Science, and The Cochrane Library) and Chinese database (CNKI, Wanfang, Weipu (VIP), CBM). Besides, manually search for Google and Baidu academic of compound XuanJu capsule combined western medicine in the treatment of male oligoasthenotspermia in randomized controlled clinical research. The retrieval time limit was from the establishment of the database to July 2020. Two researchers independently extracted and evaluated the quality of the data in the included study. A meta-analysis was performed using RevMan5.3 software, no language restrictions.

**Results:**

In this study, the efficacy and safety of compound Xuanju capsule combined with western medicine in the treatment of male oligoasthenotspermia were evaluated by the total effective rate, semen parameters and other indexes.

**Conclusions:**

This study will provide reliable evidence-based evidence for the clinical application of compound Xuanju capsule combined with western medicine in the treatment of male oligoasthenotspermia.

**Ethics and dissemination:**

Private information from individuals will not be published. This systematic review also does not involve endangering participant rights. Ethical approval was not required. The results may be published in a peer-reviewed journal or disseminated at relevant conferences.

OSF Registration number: DOI 10.17605/OSF.IO/2PM8T.

## 1 Introduction

According to the statistics of the World Health Organization (WHO), the incidence of infertility among married couples of childbearing age is about 15%, while the male factor accounts for about 40%.^[1]^ Lack of sperm and weak sperm are the main causes of male infertility. Routine semen examination was performed on male patients for 2 consecutive times. The results of the 2 examinations indicated that sperm density was less than 20 × 10^6^/ml, and the sum of the forward motile A-grade sperm and B-grade sperm, namely A-grade + B-grade sperm <50% or A-grade sperm <25%, could be diagnosed as oligoasthenotspermia.^[2]^ According to western medicine, its pathogenesis is related to testicular dysfunction, varicocele, excessive oxidative stress,^[3]^ environment, diet, genetic genes,^[4]^ stressful life and work, sexually transmitted diseases and other factors.^[5]^ For the time being, there is no specific drug for the treatment of male spermatogenic disorders in modern medicine. Although the existing surgical and hormone drugs are effective, they have a narrow range of adaptation. Assisted reproductive technology has solved the problem of reproductive difficulties to a great extent, however, there are many disadvantages, such as low live birth rate, huge cost, many side effects and ethical risks.^[6]^

Compound Xuanju capsule is a kind of Chinese patent medicine, which is mainly composed of black ants, plus Herba Epimedii, Fructus Lycii, and Fructus Cnidii. It is reasonably compatible according to the theory of traditional Chinese medicine.^[7]^ According to traditional medicine, compound Xuanju capsule has the effects on warming kidney, invigorating Yang, benefiting kidney essence and dispelling rheumatism, and can be used to treat oligoasthenotspermia in male infertility.^[8]^ Traditional Chinese medicine has accumulated rich experience in the treatment of male infertility and oligoasthenotspermia, and the curative effect has been proved.^[9]^ Individualized treatment can be provided according to the patient's condition. Compound Xuanju capsule can improve male semen quality and male reproductive function by adjusting human Yin and Yang, Qi and blood, and it is simple, safe and effective, with few adverse reactions and no drug dependence.^[10,11]^

At present, many clinical studies have shown that compound Xuanju capsule combined with western medicine is effective in the treatment of male oligoasthenotspermia with high cure rate, low recurrence rate and few adverse reactions, but the number of clinical trials is less, and also there are differences in research design and efficacy, which affect the promotion of this therapy to a certain extent. Therefore, in this study, we conducted a meta-analysis to explore the effects of compound Xuanju capsule combined with western medicine on the reproductive ability and quality of life of patients with oligoasthenotspermia, so as to provide a reliable evidence-based basis for the treatment of male oligoasthenotspermia by compound Xuanju capsule combined with western medicine.

## 2 Methods

### 2.1 Protocol register

This protocol of systematic review and meta-analysis has been drafted under the guidance of the Preferred Reporting Items for Systematic Reviews and Meta-Analysis Protocols (PRISMA-P). Moreover, it has been registered on the open science framework (OSF) on September 10, 2020. (Registration number: DOI 10.17605/OSF.IO/2PM8T).

### 2.2 Ethics

Since this is a protocol with no patient recruitment and personal information collection, the approval of the ethics committee is not required.

### 2.3 Eligibility criteria

#### 2.3.1 Types of studies

We collected all available randomized controlled trails (RCTs) on compound Xuanju capsule combined with western medicine in the treatment of male oligoasthenotspermia, regardless of blinding, publication status, region, but Language will be restricted to Chinese and English.

#### 2.3.2 Object of studies

Patients with oligoasthenotspermia were clearly diagnosed according to the WHO, Standard Diagnostic Manual for Infertile Couples, and The Chinese Guidelines for Clinical Research on New Chinese Medicines. There were no other complications. There were no restrictions on nationality, race, age, gender, course of disease and location of the included samples.

#### 2.3.3 Types of interventions

The treatment group: use compound Xuanju capsule only or combined with western medicine.

The control group: western medicine intervention or placebo.

#### 2.3.4 Types of outcome indicators

1. Main outcome: Effective rate, Pregnancy rate;
2. Secondary outcome: Sperm density, sperm motility, number of Grade A sperm, percentage of normal sperm morphology.

### 2.4 Exclusion criteria

1. Studies published repeatedly;
2. Studies whose literature are abstract and conference papers, in which the original data cannot be obtained;
3. Studies whose data are incomplete or where there are obvious errors that cannot be handled after contacting the author;
4. Studies with wrong random method;
5. Studies in which subjects with infertility caused by uncured genital infection, sex hormone disorder and other reasons.
6. Studies in which subjects were given drugs that inhibit sperm production and sperm motility.

### 2.5 Search strategy

“Oligospermia”(shao jing zheng), “ asthenotspermia”(ruo jing zheng), “male infertility”, “compound Xuanju capsule”(fu fang xuan ju jiao nang) and other Chinese search terms were used for retrieval in Chinese databases, including CNKI, Wanfang Data Knowledge Service Platform, VIP Information Chinese Journal Service Platform, and China Biomedical Database. English retrieval words such as “ oligoasthenotspermia”, “oligospermia”, (“Compound Xuanju Capsule” were used for retrieval in English databases, including PubMed, EMBASE, Web of Science and the Cochrane Library. In addition, manual retrieval was performed in Baidu and Google academic. The retrieval time was from the establishment of the database to July 2020, and all the domestic and foreign literatures about compound Xuanju capsule combined with western medicine in the treatment of male oligoasthenotspermia domestic and foreign literatures on the treatment of were collected. Take PubMed as an example, and the retrieval strategy is shown in Table 1.

**Table 1:**
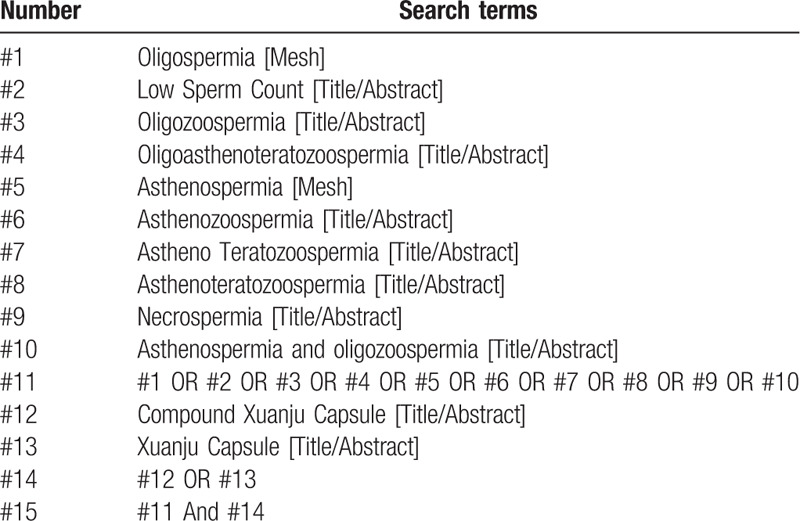
Search strategy in PubMed database.

### 2.6 Data screening and extraction

Cochrane Collaboration System Reviewer Manual Version 5.0 was used as a reference for the method of selection in the study. According to the PRISMA flow chart, EndNote X7 document management software was utilized by 2 researchers to independently screen the documents based on the above inclusion and exclusion criteria before mutual check. Those difficult to determine whether included in the study, would be discussed and judged with a third researcher. At the same time, Excel 2013 was used to extract relevant information, including:

1. Clinical features (title, first author, publication year and month, sample size, sex ratio, average age, average course of disease);
2. Intervention measures: The dosage, frequency and course of treatment of compound Xuanju capsule, the name, frequency and course of treatment of western medicine used in the treatment group, the name, administration mode, frequency and course of treatment of western medicine used in the control group;
3. Evaluation factors of risk bias in randomized controlled studies;
4. Observation indicators.

**Figure 1:**
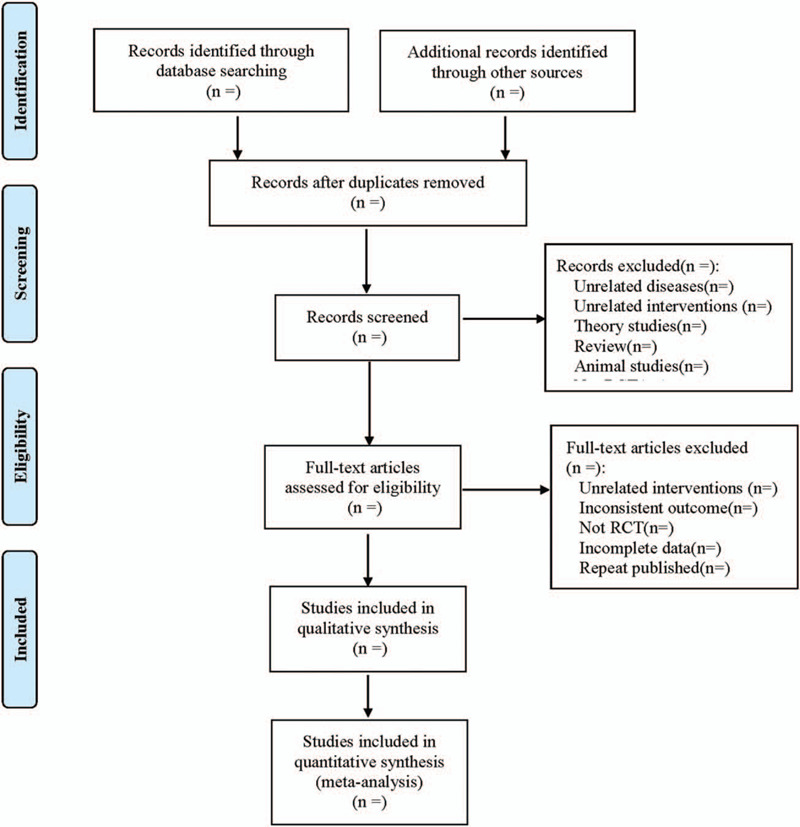
Flow diagram.

The literature screening process is shown in Figure 1.

### 2.7 Literature quality evaluation

Built-in Risk bias evaluation tool of Review Manager 5.3 Software (the Cochrane collaborations tool for assessing risk of bias) was used to assess the risk bias in the included studies. Two researchers determined the literatures from 3 levels, including low-risk, unclear, and high-risk based on the performance of the included literature in the above evaluation items. After completion, they would recheck. In case of a disagreement, they would discuss. If no agreement could be reached, a decision would be made in consultation with researchers from the third party.

### 2.8 Statistical analysis

#### 2.8.1 Data analysis and processing

The RevMan 5.3 software provided by the Cochrane Collaboration was used for statistical analysis.

1. For dichotomous variables, relative risk (RR) was used for statistics. For continuous variables, weighted mean difference (WMD) was selected when the tools and units of measurement indicators are the same, standardized mean difference (SMD) was selected with different tools or units of measurement, and all the above were represented by effect value and 95% confidence interval (CI).
2. Heterogeneity test: Q test was used to qualitatively determine inter-study heterogeneity.

If *P* ≥ .1, there was no inter-study heterogeneity, if *P* < .1, it indicated inter-study heterogeneity. At the same time, *I*^2^ value was used to quantitatively evaluate the inter-study heterogeneity. If *I*^*2*^ ≤ 50%, the heterogeneity was considered to be good, and the fixed-effect model was adopted. If *I*^*2*^ > 50%, it was considered to have significant heterogeneity, the source of heterogeneity would be explored through subgroup analysis or sensitivity analysis. If there was no obvious clinical or methodological heterogeneity, it would be considered as statistical heterogeneity, and the random-effect model would be used for analysis. Descriptive analysis was used if there was significant clinical heterogeneity between the 2 groups and subgroup analysis was not available.

#### 2.8.2 Dealing with missing data

If data is missing or incomplete, we will contact the corresponding author to obtain the missing data. If not, this study will be removed.

#### 2.8.3 Subgroup analysis

Subgroup analysis was carried out according to the treatment group of compound Xuanju capsule combined with western medicine and Chinese herbal compound treatment only. Subgroup analysis was carried out according to the age of the patients, which can be divided into 2 subgroups: young people and middle-aged people. Subgroup analysis was carried out according to the disease, which can be divided into 2 subgroups: male oligospermia and male asthenospermia. Subgroup analysis can also be performed according to the course of treatment.

#### 2.8.4 Sensitivity analysis

In order to test the stability of meta-analysis results of outcomes, a one-by-one elimination method will be adopted for sensitivity analysis.

#### 2.8.5 Assessment of reporting biases

For the major outcome indicators, if the included study was ≥10, funnel plot was used to qualitatively detect publication bias. Eggers and Beggs test are used to quantitatively assess potential publication bias.

#### 2.8.6 Evidence quality evaluation

The Grading of Recommendations Assessment, Development, and Evaluation (GRADE) will be used to assess the quality of evidence. It contains 5 domains (bias risk, consistency, directness, precision, and publication bias). And the quality of evidence will be rated as high, moderate, low, and very low.

## 3 Discussion

The understanding of male infertility in traditional Chinese medicine has a history of more than 2000 years, and there are many related discussions in ancient books of traditional Chinese medicine.^[12]^ Traditional Chinese medicine believes that the kidney stores essence and dominates reproduction, and the production of spermatozoa is directly related to the kidney. If the kidney is deficient in producing nutrients, sperm production will be inadequate; the spleen is the acquired foundation and the source of Qi and blood, and also can transport nutrient substance in the body to nourish the kidney essence so as to transform into reproductive essence.^[13]^ Lack of congenital endowment and nurturing, sexual immoderation, irregular diet, emotional discomfort and other factors will consume kidney essence and cause kidney essence deficiency. Then sperm becomes scarce, semen becomes thin, resulting in male oligospermia and oligoasthenotspermia.^[14]^ Modern studies have also shown that oligoasthenotspermia patients with kidney deficiency infertility are different from normal men in plasma metabolic pathway and biomolecule level, which may be the cause of oligoasthenotspermia.^[15]^

Compound Xuanju capsule mainly contains Black Ants, Herba Epimedii, Fructus Cnidii, and Fructus Lycii. In the compound Xuanju capsule, the major medicine is the Black Ant, assisted by Herba Epimedii, both of which belong to the liver and kidney meridians. The capsule has the effect of tonifying the kidney essence and activating collaterals.^[16]^ Using Fructus Lycii and Fructus Cnidii to warm the kidney, tonify essence, strengthen tendons and tonify Yang, can significantly improve the symptoms such as deficiency of kidney Yang, lethargy, fatigue, cold sperm and night emission, low sexual desire and so on. Modern medical research has found that Black Ants, Epimedium and other drugs are rich in amino acids, vitamins, coenzymes, flavonoids and other ingredients,which can significantly increase the content of various nutrients in the human body.^[17]^ Clinical studies have shown that compound Xuanju capsule can effectively improve semen quality and reduce sperm chromatin damage in infertile men with oligoasthenotspermia;^[18]^ increase the level of sex hormone, promote semen secretion, improve the function of sexual organs;^[19]^ significantly increase the concentration and total number of sperm, increase sperm motility;^[20]^ effectively improve the semen quality and sperm morphology of infertile male smokers; reduce genital inflammation and regulate sexual dysfunction.^[21]^ However, the intervention of Chinese patent medicine lacks the process of syndrome differentiation and treatment in traditional Chinese medicine, it is difficult to give full play to the best effect of traditional Chinese medicine treatment. Moreover, the deficiency syndrome of traditional Chinese medicine pays attention to slow conditioning and the treatment usually requires a long course of treatment.

At present, many trials of compound Xuanju capsule in the treatment of male oligoasthenotspermia have been widely reported, but there is a lack of systematic evaluation. Therefore, it is necessary to objectively evaluate the effect of compound Xuanju capsule on patients with oligoasthenotspermia through evidence-based medicine, promote the treatment of compound Xuanju capsule, and provide scientific and evidence-based prescription of traditional Chinese medicine for clinic. However, this study also has some limitations, such as the lack of large samples and high-quality randomized controlled trials. What's more, it is difficult to evaluate the long-term efficacy of drugs caused by short follow-up time. At the same time, due to the limitation of language ability, only English and Chinese literature are searched, the research in other languages may be ignored. And there also may exist some publication bias.

## Author contributions

**Data collection:** Lili Chen, Hongyi Lan.

**Funding support:** Yu Liu.

**Literature retrieval:** Lili Chen, Hongyi Lan.

**Software operating:** Yuyan Zhang, Xiaoyan Zhang.

**Supervision:** Yu Liu.

**Writing – original draft:** Lili Chen, Hongyi Lan.

**Writing – review & editing:** Lili Chen, Yu Liu.
